# Progress of Veterinary Science

**Published:** 1884-01

**Authors:** 


### Progress of Veterinary Science.

AT THE LAST MEETING of the American Public Health Association, held at Detroit, Mich., the officers for the next year were elected as follows:

President, Dr. Albert L. Gihon, U. S. Navy.
First Vice-President, Dr. James E. Reeves, Wheeling, W. Va.
Second Vice-President, Hon. Erastus Brooks, New York.
Secretary, Dr. Irving A. Watson, Concord, N. H.
Treasurer, Dr. J. Berrien Lindsley, Nashville, Tenn.
Executive Committee, Dr. Thos. L. Neal, Ohio; Dr. J. D. Gatch, Indiana; Dr. H. P. Walcott, Mass; Dr. Gustav Devron, La: Dr. Chas. Smart, U. S. Army; Dr. H. D. Fraser, S. C.

HOG CHOLERA.—Dr. J. M. Partridge, of South Bend, Ind., read a report on hog cholera, giving its history. He stated that it was a disease sui generis, having distinct pathological characteristics in all localities. He described the bacillus suis, claimed to have been discovered by Detmars. The infectious principle may be introduced into the system by food or drink, or through cuts or abrasions. As preventive measures all cuts or bruises should be kept closed by applications of tar, or some substance imprevious to air or water; water should be pure and cars clean, and carbolic acid in ten drops to each one hundred weight of the animals may be fed to the hogs, three times per day. The sick hogs should be removed from the well, or destroyed. Separate the well into small herds, and remove them to fresh pastures.

TEXAS CATTLE FEVER.—D. E. Salmon, V. S., read a paper on Texas cattle fever, in which he states that the disease known by that name was present constantly in several of the southern states, is infectious, and that one mode of its spread, at least, is through pastures and infective excreta of affected cattle, and that the disease might be carried in this way among cattle to distant states. He stated that the Texas cattle, on their native ranges, were almost invariably afflicted with the fever. If northern cattle were to be saved from this contagious disease, a quarantine must be declared against Texas cattle. Among the thousands of cases in the north, there was not one which could not be traced to cattle from that state.

The paper was discussed by Dr. Regan, of Texas, and others.—*Sanitary News.*

ACTINOMYCOSIS DISCOVERED IN AMERICAN CATTLE.—Dr. William T. Belfield, of Chicago, has made the important discovery that actinomycosis exist in American cattle. He was asked by the Commissioner of Health of Chicago to investigate a disease in cattle which has generally been known as "swell-head," and has been called by veterinarians cancer, sarcoma. etc. Five animals were examined by Dr. Belfield, and a very short study of the specimens under the microscope revealed the true nature of the disease. Actinomycosis was only recognized six years ago by Böllinger, of Munich, who announced that it was a parasitic disease due to the presence of a rapidly growing fungus. It has since been discovered in the hog and in man. It generally first attacks the jaws, and probably gains access to the deeper tissues through carious or defective teeth. It spreads into the tissues of the head, causing tumefactions, suppuration, finally, if unchecked, pyæmia, and death. It may gain the blood and be transferred to other parts of the body. This happens especially with man, upon whom the parasite acts most virulently. It is supposed that its source is the grain with which animals are fed. The disease is generally fatal, though prompt measures may check it. The meat of animals dying from actinomycosis is not of first quality. It is not, however, yet known that it is absolutely injurious. Thorough cooking, at any rate, destroyes the parasite. Dr. Belfield's discovery is an important one, and should become promptly known to veterinarians and sanitary officials.—*Medical Record.*

PROPAGATION OF DIPHTHERIA BY CHICKENS.—It has been known for some time that pheasants, pigeons, turkeys, domestic fowl, and the like were liable to be attacked by diphtheritis. The *Wiener Allgemeine Medicinische Zeitung* informs us that Prof. Gerhardt of Würzburg has carried out a series of observations for the purpose of determining whether the disease may be communicated by this means, and has come to an affirmative conclusion. In September, 1881, 2,600 fowls were sent from the neighborhood of Verona to Nesselhausen, in Baden, where there is a great fowl-rearing establishment. Some of them must have been affected with diphtheritis before they started, and in the end 1,400 fowls died of it. In the summer of last year 1,000 chickens were hatched from eggs collected from many different places. Six weeks after their birth diphtheritis manifested itself among the young chickens, and so badly that in a short time they all died. Five cats that were kept in the establishment also became ill of the same malady and died. A parrot that hung in a cage in the house was also attacked, but recovered.

Last November an Italian hen, which had been "painted" about the jaws with carbolic acid by the keeper, bit the man's wrist and foot. Presently he became ill with a smart fever, considerable swelling at the wounded parts, and all the symptoms of traumatic diphtheritis. His recovery was very tedious. This was not the only case of the transmission of the disease to men. Two-thirds of all the laboring persons employed about the establishment became ill with ordinary diphtheritis, and one man conveyed the infection to his three children. It is worth noting that during all this time no other diphtheritic cases occurred at Nesselhausen, or in the neighborhood. The inference seems obvious that all these cases originated with the sick fowls.—*London Times.*

THE CONTAGION OF TUBERCULOSIS.—The following are the conclusions arrived at by M. Daremberg in a communication addressed to the Academy of Medicine of Paris (*Bulletin de l'Académie*, October 7, 1883): 1. Tuberculosis is a disease of parasitic nature, transmissible by inoculation, alimentation, or inhalation. 2. It is always caused by the absorption of a germ from without. 3. When occuring by inhalation the quantity of the contagion is of little moment. It can operate only in a suitable soil. In a word, contagion is an influence to which all are exposed, but which is operative only in those individuals in whom hereditary or acquired vices of nutrition have prepared a field suited to the growth and reproduction of the germ. 4. Local and general tuberculoses differ only in degree. 5. Scrofula appears to be a diathesis, while tuberculosis is an infection engrafted upon diatheses. 6. The infectious germs of tuberculosis are not usually inherited, but rather the vices of nutrition which have provoked the disease in the ancestors. 7. Therapy, while permitted to seek for specifics, ought, in the way of prophylaxis, to aim at the destruction, through general hygiene, of the causes which prepare a fit soil for the reception of the tuberculous contagion. After the disease is established it should seek to combat the phenomena arising in the economy from the presence and proliferation of the infectious agent.—*Medical Record.*

DIARRHŒA IN A CAMEL.—While visiting a sick elephant at the cattle-karkhana I happened to see a camel evidently in the last stage of exhaustion. He had been suffering a long time from diarrhœa and died the next day. I made a careful post-mortem examination and found hydaties in both liver and lungs. The lining membrane of the true stomach was everywhere studded with small globular yellow deposits, equal in size to an average mustard seed; a remarkable pathological state of which I have never observed another instance. (I availed myself of this opportunity to determine exactly what is the part protruded from the mouth of the camel with a gurgling sound at the period of rutting. It is a loose sac of highly glandular mucous membrane depending from the antero inferior surface of the fixed and pendulous portions of the enormously developed soft palate. —*J. H. Steel, V. S. in the Quarterly Journal of Veterinary Science in India.*

Pneumocele in a Cow.—Removal of a portion of the right lung. Recovery. On the 24th of July of the current year I was requested to see a cow, which, as related by the owner, had been gored in the chest the day before, by one of the oxen. A tumefaction of the part followed, which originally the size of a nut gradually increased.

On examination, I found the animal to be in a perfectly normal condition with the exception of the thoracic region, where, on the right side about 20 cm. from the elbow at a point corresponding to the 6th intercostial space, a lacerated contused wound existed. It was almost round, 2 cm. in diameter, and from it exuded a sero sanguineous fluid. This was followed by the protrusion of a tumor at the 9th intercostal space, corresponding, doubtless, to the internal wound. This tumor was painful and hard to the touch, and elastic only at the point of termination.

The external wound presented at its deepest part a smooth, movable, isolated body of a dark red color.

All attempts at reduction by taxis failed. As the inflamatory action was found by auscultation to be limited to the protruded portion of the lung, I determined to remove it at a point corresponding to the internal wound. Having cast the animal, I made an incision from the external wound to a point on the skin corresponding to the internal one, opened the hernial sac, and, placed a ligature around the protruded lung substance. Antiseptic dressings and bandages where then applied, and on the 10th day the tumor sloughed off. One month from the date of operation the animal was entirely well.

The importance of the organ involved and the rarity of the case induced me to publish it with the hope that it may prove of interest to my colleagues and useful to our science.—*Dr. Giovanni Guglielmi, in La Clinica Veterinaria.*

Acute Gastro-enteritis in a Bear.—At a meeting of the Pathological Society of London, Mr. J. Hutchinson, Jr., showed the stomach of a female bear which had been at the zoölogical gardens for about four years, and died in February last after one days illness. The stomach and upper part of the intestines were acutely inflamed; the rest of the alimentary canal was healthy; There was no food, but much exudation in the stomach and neighboring parts of the intestines. No torulæ, sarcinæ, or micro-organisms were found, but minute quantities of round cells and fibrin, with altered blood. The uterus, bladder, and trachea, showed acute catarrh. The other viscera were healthy. Probably the disease was purely catarrhal It was noteworthy that there was no fur whatever on the tongue.—*The Veterinarian.*

Medicine as Practiced by Animals.—J. H. Steel, V. S. in the "Quarterly Journal of Veterinary Science in India," states that the elephant when affected with amphistomes voluntarily eats earth to act as a mechanical cathartic and vermifuge. In fever he throws cold water over his body. When his foot is inflamed he irrigates it with the trunk, and the natives of Ceylon state that elephants plug up bullet holes with clay.

Posology and Therapeutics.—Fred. Smith, V. S. in the "Quarterly Journal of Veterinary Science in India," was led to examine the doses of different pharmocopœial agents by the following incident: A horse that was under our care was deemed incurable, and the owner being willing to have it destroyed, we determined to poison him. The agent selected was tincture of aconite, which if given in a large dose should cause instaneous death. Half an ounce was given, followed in two hours by three ounces, and in seven hours by fourteen ounces but without producing the slightest effect. On the following day one and a half pints were administered with the same result. We next gave at intervals six ounces of tincture of digitalis and one pint of tincture of opium. The animal manifested not the least drowziness and the next morning was found to be as well as ever. We then bled him to syncope and gave three ounces of indian hemp. The effect was most marked; he slept all day and became partially comatose. Recovering again, in despair we administered three ounces of hydrocyanic acid, and this having no effect, the animal was finally destroyed by bleeding.

In this experiment we gave enough aconite for 1,140 horses, digitalis for 6, and opium and indian hemp for 12. We may well call these results startling, and may be excused for doubting the correctness of our doses, or even the efficacy of our agents.

We believe that aconite is non-poisonous. and has no therapeutic action on the horse, and that opium is very doubtful as an anodyne. We cannot condemn digitalis; it is a cumulative drug and takes some time to act; six ounces is evidently not a poisonous dose. Cannabis Indica is the only agent we place any faith in as a sedative and anodyne. It was this experiment which first opened our eyes to its virtues,and we have used it since then and have never been disappointed; its dose is from one to one and one-half ounces. If given in the usual drachm doses, it is sure to fail and disappoint.

Inoculation with Virus of Farcy-glanders.—*1st experiment.* Female donkey, small. Inoculation of near nostril and under tail by scarification,virus from nostril of a horse: also 80 minims of blood from jugular of horse injected hypodermically under chest. Four days after a scab and slight swelling at seats of inoculation.

*Fifth day*: pulse 68. temp. 101.2° F. dull and off feed *Sixth day*: pulse 76, temp. 101° F. *Eighth day*: some fever. *Tenth day*: a soft, hot and tense swelling on off cheek. *Twenty-sixth day*: no loss of health, swelling has disappeared, seat of inoculation under the tail discharging a small amount of pus. *Thirty-third day*: a soft "bud" inside near forearm. *Thirty-fourth day*: the bud burst, the temperature ranging between 99° and 101° F. *Fifty-second day*: an enlargement on the chest and bubœs forming in the groin. *Sixty-second day*: shot; autopsy: groups of ulcers in near nostril, many buds in mesentery. Lungs studded here and there with inflamed patches. One of the lymphatic glands internally to the shoulder filled with pus.

*2nd experiment.* Entire donkey, small. Inoculation of off nostril and under side of tail with virus from a sore on near hind-limb of horse. Also two endermic injections of a mixture of virus from a freshly opened bud with three times its weight of water inside near thigh and off forearm. Also four drachms of blood taken from the jugular injected into the tissues of near side of neck.

*Second day*: temp. 98°, pulse 48. *Third day*: temp. 101°, pulse 52. *Fourth day*: temp. 100°, pulse 53; a swelling observed behind left shoulder, in the evening temp. 102°, pulse 72, respirations quickened, patient very uneasy, off feed, dull and frequently lies down. *Fifth day*: a little better, but mucous membranes highly injected. *Sixth day*: temp. 101.2°, pulse 70, buds forming on chest. swelling behind shoulder has burst. *Ninth day*: every symptom aggravated. The patient looks the picture of misery and buds are forming rapidly along the inside of the thighs and other places. Shot: no autopsy.

*3d experiment.* Female donkey. Inoculated with a solution, one to three, of the virus from a fresh ulcer with warm water, injected hypodermically inside of thigh and behind off shoulder. *Fourth day*: temp. 94 to 100°, patient dull. *Fifth day*: temp. 102°, pulse 72, completely off feed, dull and listless, slight swelling inside near hind limb, also a small sore on near side of nose. *Tenth day*: a swelling on off forearm and under shoulder. *Fourteenth day*: swelling has burst and is discharging. *Sixteenth day*: discharge ceased, swelling smaller. *Twentieth day*: swellings inside both thighs. *Thirtieth day*: all swelling have subsided, but a few sluggish hard buds are left. *Fiftieth day*: swellings have returned. *Fifty-first day*: true farcy buds have formed in the groins and one under the tail. *Fifty-second day*: temp. 102°. *Sixty-third day*: shot: autopsy showed bubœs in groins, ulceration of near nostril, heart infested with tubercle, mesenteric glands enlarged and bud-like. Lungs discolored and here and there buds in a state of formation, and at the base of the organs a cluster of yellowish green buds (bronchial glands?) containing a quantity of greenish cheesy looking matter.

(Mr. Mills records as a noteworthy fact, that he had observed the average temperature of country donkeys in health to be only 94.2° F.)—*Veterinary Surgeon Mills on the Quarterly Journal of Veterinary Science in India.*

Syphilitic Inoculation of Monkeys.—At a recent meeting of the Société Médicale des Hôpitaux, Dr. Martineau announced that the monkey he inoculated on November 16, 1882, with syphilitic matter from a patient of his, after having presented the characteristic lesions—hard chancre, followed by the various syphilides (papuloerosive)—became affected ten months after inoculation with ulcerous syphilide of the mucous membrane of the palate, which has healed. This case is interesting in so far as it proves that the evolution of syphilis in this monkey is following the usual course observed in man, and will tend to affect the theories in vogue respecting the natural history and origin of syphilis.—*Medical Record.*

Bone Disease in Monkeys.—At a meeting of the Pathological Society of London, Mr. Sutton said that the most prominent symptoms of rickets in a monkey were diminished activity, paralysis of the lower limbs, so that the animal dragged himself along, using his arms as crutches, which caused these to bend; gradually the paraplegia became complete; there was priapism incontinence of urine and feces. The disease ran a very rapid course, and the animal died in three or four months' time from bronchitis or broncho-pneumonia. In capuchins the chief signs were beaded ribs, softened and curved bones, enlarged epiphyses, deformed pelves; the skull was remarkably eroded, perforated and slightly thickened; on either side of the foramen magnum was a tabetic patch. The shafts of the long bones presented a most remarkable condition, the compact tissue being split up into longitudinal lamellœ, separated by tracts of richly cellular connective tissue, which was readily seen to be continuous with the deeper layers of the periosteum; the medullary cavities were filled with dark red marrow; the epiphyses showed the condition he had already described as diffuse epiphysis. and he was satisfied that the enlarged epiphysis met with in rickets were due to the ossific matter being deposited in a diffuse and irregular manner. The paraplegia was due to compression of the spinal cord from overgrowth and softening of the vertebra gradually encroaching upon it. The nerves also, partly from the same cause, and partly from the weight of the body, would get pinched in the intervertebral foramina, and thus he would explain the pains in the legs and urino-genital troubles which formed such prominent symptoms in the disease. Examination of the spinal cord with the microscope showed all the changes found in the cord in compression from cancer, Pott's disease, etc.

Dr. Goodhart asked for some information relative to the age of these monkeys. Last Session Mr. Sutton had shown these changes in animals about the age of four years; and if these animals were as old, the disease was probably more allied to "late rickets" in the human subject than to ordinary rickets, and therefore must be regarded as a totally distinct disease. He also asked if lardaceous disease had been present in any of these animals.

Mr. Sutton, in reply, said it was exceedingly difficult to estimate the age of the monkeys; they were not born in the gardens, and the state of the epiphyses was almost the only guide, and that not a very reliable one. He had not found lardaceous disease again since the case reported last session.—*The Veterinarian.*

